# Interventions and practices using Comfort Theory of Kolcaba to promote adults’ comfort: an evidence and gap map protocol of international effectiveness studies

**DOI:** 10.1186/s13643-023-02202-8

**Published:** 2023-03-06

**Authors:** Yanxia Lin, Yi Zhou, Can Chen

**Affiliations:** 1 School of Nursing, Shanghai University of Traditional Chinese Medicine, NO. 1200, Cailun Road, Pudong District, Shanghai, 201203 China; 2 School of Nursing, Langfang Health Vocational College, Siguang Road, Guangyang District, Langfang, Hebei 065000 China; 3 School of Nursing, Hebei University of Chinese Medicine, NO. 3, Xingyuan Road, Luquan District, Shijiazhuang, Hebei 050200 China

**Keywords:** Comfort care, Comfort intervention, Comfort measurement, Comfort Theory, Evidence and gap map, Mapping review, Patient comfort

## Abstract

**Background:**

Comfort is a primary patient objective and central to patient experience, and thus, maximising comfort is a universal goal for healthcare. However, comfort is a complex concept that is difficult to operationalise and evaluate, resulting in a lack of scientific and standardised comfort care practices. The Comfort Theory developed by Kolcaba has been the most widely known for its systematisation and projection and most of the global publications regarding comfort care were based on this theory. To develop international guidance on theory-informed comfort care, a better understanding about the evidence on the effects of interventions guided by the Comfort Theory is needed.

**Objectives:**

To map and present the available evidence on the effects of interventions underpinned by Kolcaba’s Comfort theory in healthcare settings.

**Methods:**

The mapping review will follow Campbell Evidence and Gap Maps guideline and Preferred Reporting Items for Systematic Reviews and Meta-Analyses extension for Scoping Reviews Protocols guidelines. An intervention-outcome framework has been developed based on Comfort Theory and the classification of pharmacological and non-pharmacological interventions via consultation with stakeholders. Eleven electronic databases (MEDLINE, CINAHL, PsycINFO, Embase, AMED, Cochrane Library, JBI Library of Systematic Reviews, Web of Science, Scopus, CNKI and Wan Fang) and grey literature sources (Google Scholar, Baidu Scholar and The Comfort Line) will be searched for primary studies and systematic reviews between 1991 and 2023 written in English and Chinese as the papers regarding Comfort Theory were first published in 1991. Additional studies will be identified by reference list review of included studies. Key authors will be contacted for unpublished or ongoing studies. Two independent reviewers will screen and extract data using piloted forms with discrepancies resolved by discussion with a third reviewer. A matrix map with filters of study characteristics will be generated and presented through software of EPPI-Mapper and NVivo.

**Discussion:**

More informed use of theory can strengthen improvement programmes and facilitate the evaluation of their effectiveness. Findings from the evidence and gap map will present the existing evidence base for researchers, practitioners and policy-makers and inform further research as well as clinical practices aiming at patients’ comfort enhancement.

**Supplementary Information:**

The online version contains supplementary material available at 10.1186/s13643-023-02202-8.

## Background

### Introduction

#### The problem, condition, or issue

Comfort is a primary patient objective and central to patient experience [1, 2], and thus, maximising comfort is a universal goal for healthcare [3, 4]. Comfort is a pleasant experience, a desired state of satisfaction [5] and feeling positive and strengthened in one’s ability to cope with crisis and challenges [6]. Enhanced comfort after therapeutic interventions may increase hope and confidence and facilitate healing, rehabilitation and dying peacefully [4, 5, 7].

Comfort, however, is a complex concept that is difficult to define, operationalise and evaluate. Comfort experience is transient and multidimensional and corporates more than the absence of pain [2]. It is a dynamic concept, varying and individualised [5], with inherent properties of change over a period of time, for example 20-min time frame [8, 9]. There are many meanings of the term and no consensual definition has been achieved [10–12]. The literature shows comfort is used in different ways, as a noun, a verb, an adjective and also as a state, a process and an outcome [5]. Studies about the analysis of the concept continue to be developed even today [10]. Without a unified definition, its measure is an issue resulting in the difficulty in assessing comfort needs and a limitation to studies testing of the efficacy and effectiveness of comfort interventions. Evidence shows that nurses had difficulties to assess the patient to fulfil their comfort needs calling for practical yet valid and reliable comfort assessment tools [13]. More essentially, it remains unclear on determining effective and efficient interventions to enhance comfort and specific outcomes and consequences of comfort interventions are rarely documented [5].

#### The intervention

Comfort has not been fully conceptualised and operationalised until American researcher Dr. Katherine Kolcaba developed the Comfort Theory [4, 14, 15]. Comfort Theory is a mid-range theory developed in the context of elderly care, home care and long-term care in the USA. Among the different theorists, Kolcaba’s Comfort Theory is the most widely known for its systematisation and projection [16] and the majority of the publications regarding comfort were based on her comfort theory [10].

**Fig. 1:**
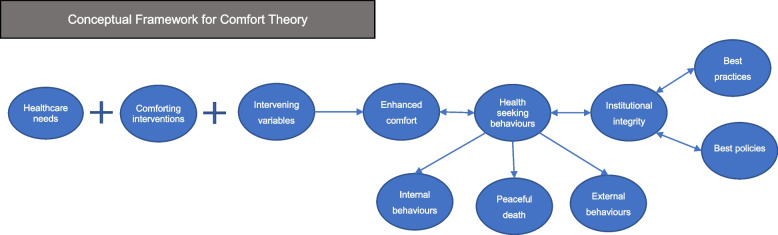
Conceptual framework for Kolcaba's Comfort Theory: Healthcare needs—comfort needs in physical, psychospiritual, environmental and social-cultural contexts; Comforting interventions—therapeutic interventions to enhance comfort; Intervening variables—factors that HCPs cannot easily change (i.e. extent of social support, financial resources); HSBs—internal (i.e. healing), external (i.e. self-care activities) and a peaceful death; Institutional integrity—the value, financial stability and wholeness of health care organisations at the local, regional, state and national levels

The conceptual framework of Comfort Theory [17] is depicted in Fig. 1, which proposes that:

1. Healthcare professionals (HCPs) assess comfort needs of patients/family members that are not met.
2. HCPs design interventions to address those needs.
3. Intervening variables are taken into account in designing interventions.
4. HCPs measure comfort before and after interventions
5. If comfort is enhanced, patients/families engage more fully in health-seeking behaviours (HSBs).
6. When HSBs are enhanced, the institution integrity is enhanced.

Intervening variables are those factors that HCPs cannot easily change, such as extent of social support and financial resources [1]. HSBs involve internal behaviours, external behaviours and peaceful death. Internal behaviours occur at the cellular or organ level, such as healing while external HSBs concern the outer world, such as self-care activities. Institutional integrity is the value, financial stability and wholeness of health care organisations at the local, regional, state and national levels [17].

Applying the Comfort Theory in care practices is called Comfort care which is a philosophy of healthcare that focuses on addressing comfort needs, a pattern for holistic care but is individualised for each recipient or group [4]. Comfort care has three components: (a) an effective intervention, (b) a caring mode of delivery and (c) the objective to enhance comfort [4].

**Fig. 2:**
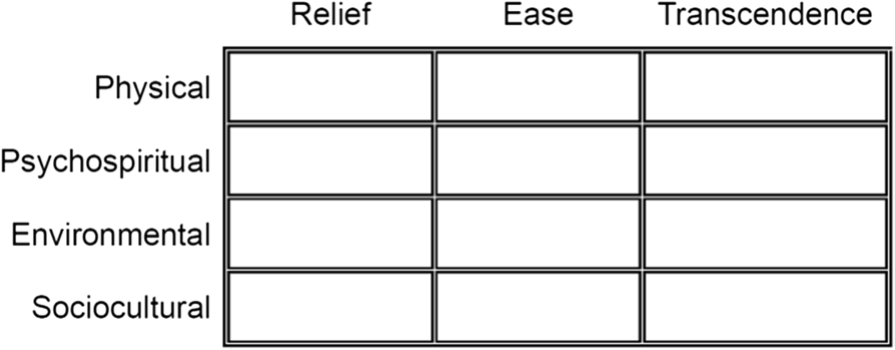
The taxonomic structure of comfort concept: type of comfort: Relief—the state of having a specific comfort need met. Ease—the state of calm or contentment. Transcendence— the state in which one can rise above problems or pain. Context in which comfort occurs: Physical—pertaining to bodily sensations and homeostatic mechanisms. Psychospiritual—pertaining to internal awareness of self, including esteem, concept, sexuality, meaning in one’s life and one’s relationship to a higher order or being. Environmental—pertaining to the external background of human experience. Sociocultural—pertaining to interpersonal, family, and societal relationships

Kolcaba defines comfort as a holistic experience: “the immediate experience of being strengthened through having the needs for relief, ease, or transcendence met in four contexts: physical, psychospiritual, environmental and sociocultural contexts” ([4] P14). Relief refers to unmet comfort needs, usually of a severe nature. Ease emphasises the importance of prevention of known risk factors that would keep a person from feeling comfortable. And transcendence indicates the ability to “rise above” discomforts when they cannot be eradicated or avoided [18]. The three types of comfort, when combined with the four contexts form a 3 × 4 taxonomic structure (TS) of 12 cells [17] (see Fig. 2). The 12 cells represent the total comfort from the perspective of patients’ needs and the fulfilment of their needs.

Holistic interventions which are meant to bring about desirable whole-person effects, such as music therapy, guided imagery [19, 20], massage [21], therapeutic touch and cognitive strategies [22, 23], can be targeted to every aspect of comfort in the TS. If an intervention developed with the TS is congruent with the outcome which is also designed with the TS, the study can demonstrate significant differences about comfort in groups [4]. Assessments of comfort before and after these interventions can demonstrate whether the interventions are effective.

Kolcaba has also developed General Comfort Questionnaire (GCQ) to measure comfort of people in general condition of illness [24], which remains the basis for many studies and the origin of other tools which are an adaptation to specific contexts and populations [5], including China [25]. For example, Hospice Comfort Questionnaire and Holistic Comfort Questionnaire were adapted from GCQ for use with patients and involved caregivers during end-of-life (EoL) care [8, 21].

#### Why it is important to develop the evidence and gap map

Comfort is a universal concept understood across most disciplines and cultures and Comfort Theory can be adopted to any healthcare settings or age group [26] as visioned by Dr. Kolcaba [17]. Since the concept analysis of comfort first published by Kolcaba in 1991 [14, 27], the Comfort Theory has been widely utilised worldwide in the past three decades [5, 10, 28]. However, there is a lack of systematic and comprehensive understanding about what evidence is there regarding whether the Comfort Theory is effective to guide interventions and practices to promote comfort. Given comfort is the primary and central goal of healthcare globally, it is essential to provide an evidence map and recognise the gaps about the effectiveness of interventions underpinned by the Comfort Theory in different contexts within an international scope.

This evidence and gap map (EGM) will collate the existing evidence and display clusters of evidence and gaps in evidence base that will serve as a resource to guide prioritisation of further research and increase the accessibility and use of evidence for informed decision-making by stakeholders including researchers, decision-makers and practitioners.

#### Existing relevant systematic reviews

To our knowledge, there is no published EGM about the effectiveness of comfort interventions utilising Comfort Theory by Kolcaba. One scoping review investigated the use of non-pharmacological interventions (NPIs) for comforting patients in palliative care, including 10 NPIs on cancer patients: aromatherapy, reiki and therapeutic touch; aromatherapy, footsoak and reflexology; aromatherapy; aromatherapy massage; massage therapy; noncontact therapeutic touch; music therapy; hypnotherapy; art therapy; and electromyography biofeedback-assisted relaxation, with three studies evaluated the total comfort and the remaining evaluated comfort related variables [28]. Another two literature reviews examined the characteristics of comfort concept [10] with one placing it in the context of paediatric critical care [29, 30]. The proposed EGM in this paper is a mapping review differing from the existing reviews by focusing on the papers reporting the application of Kolcaba’s Comfort Theory in adult healthcare. The EGM will be more comprehensive with a broader scope of all types of interventions and practices for adults. It will examine up-to-date evidence from systematic reviews as well as primary studies and map available evidence to help researchers and decision-makers make sense of the evidence available, support the creation of evidence-informed policies and guide research prioritisation.

## Objectives

The proposed EGM will map and present existing evidence on the effects of comfort interventions and practices among adults underpinned by Comfort Theory of Kolcaba in an international scope. The specific objectives of the map are to:

1. Develop a framework of types of interventions and outcomes related to the effectiveness of these comfort interventions and practices.
2. Describe the characteristics of included studies which summarise the intervention, population, country, setting and study design.
3. Identify major gaps in existing evidence base.

## Methods

The Campbell Evidence and Gap Maps guideline (EGMs) will be used to systematically search and map impact evaluation and systematic reviews on the effects of interventions and practices using Kolcaba’ s Comfort Theory to promote adults’ comfort [31]. Differing from scoping reviews and systematic reviews, EGMs provide a visual and systematic presentation of the available evidence in the form of a gap matrix for a specified sector on a broader scope, in this case “interventions using Kolcaba’s comfort theory to promote individuals’ comfort”, which show what evidence exists, rather than what evidence says [31, 32]. EGMs present the availability of data but do not synthesise the data. In line with the EGMs guideline [31, 32], the conduct and report of the mapping review will follow the Campbell EGM conduct standards checklist [33], and the Preferred Reporting Items for Systematic reviews and Meta-Analyses extension for Scoping Reviews (PRISMA-ScR) reporting checklist [34].

### Framework development and scope

Consistent with the standard EGM matrix, the current EGM will consist of two primary dimensions: rows listing intervention categories, and columns listing outcome types, and each cell of the matrix will show studies containing evidence on that particular combination of intervention and outcome. The map will also contain relevant filters, such as types of theory application, study design, population, region and country, focusing on a subset of studies meeting certain criteria. The proposed intervention-outcome framework, as described in the following, has been developed by the authors through a consultative process with stakeholders.

### Stakeholder engagement

Three international experts who were the founder and advocator of the Comfort Theory and practitioners in comfort care practices from the USA and China were consulted as the advisory panel. Katharine Kolcaba is an American nursing theorist and nursing professor. Dr Kolcaba is the founder of the Comfort Theory under investigation. She suggested a scope on the effectiveness of theory application in comfort enhancement and the criteria on descriptive articles about certain comfort interventions as well as the reliability and validity of instruments used to evaluate comfort scores. Dr April Bice is an American researcher and lecturer in paediatric nursing, and a promoter of Comfort Theory, and she offered potential sources of relevant evidence. Yongxing Shi is a Chinese associate professor and expert in community-based EoL care based in Shanghai, China, where formal palliative and hospice care services have been piloted across the city since 2017. The three experts made comments on the scope and framework of the present EGM, as well as the sources of evidence via face-to-face and email discussions.

### Conceptual framework

**Fig. 3:**
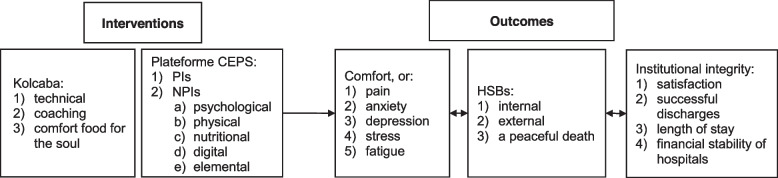
Logic model of interventions and outcomes

A matrix framework with dimensions of interventions and outcomes will be generated. Figure 3 shows the logic model for the comfort interventions and how they link to the outcomes, which is informed by the Comfort Theory of Kolcaba as outlined in Background (see Fig. 1). Comfort Theory proposes that when considering the intervening variables (age, gender, financial difficulties, cognitive impairment, etc.), if an effective intervention (hand massage) has been implemented properly it will promote individuals’ comfort experience, which can be evaluated by the comfort questionnaires [19, 21]. If individuals’ comfort is enhanced, they are strengthened to engage in HSBs including internal behaviours, external behaviours and peaceful death and engagement in HSBs further enhances comfort. Then, the enhanced comfort and engagement in HSBs will bring desired outcomes at institutional level such as satisfaction with care services, successful discharges, short length of stay and financial stability of hospitals or agencies [4]. As we work on the map, we will update and make changes to the framework if needed.

Kolcaba has proposed three categories of comfort interventions (see Table 1): (1) *Technical comfort measures* are those interventions designed to maintain homeostasis and manage pain; (2) *Coaching* is to relieve anxiety, provide reassurance and information, promote hope, listen and help plan realistically for recovery, integration, or death in a culturally sensitive way; (3) *Comfort Food for the Soul* are those comfort measures that target transcendence, making patients feel strengthened in an intangible, personalised sort of way [4, 35]. These clinical interventions can be classified into two broad categories: pharmacological and non-pharmacological interventions. According to The Collaborative University Platform for Evaluating Health Prevention and Supportive Care Programs (The Plateforme CEPS), pharmacological interventions (PIs) refer to biomedical treatments using drugs and medical devices whereas NPIs refer to science-based and non-invasive interventions that aim to prevent, care, or cure health problems [36]. Many comfort interventions such as music therapy, hand massage and guided imagery are NPIs according to the defined conditions to be NPIs [36]. The Plateforme CEPS provides a classification of NPIs which comprises five categories—psychological, physical, nutritional, digital and elemental health interventions and twenty subcategories (see Table 2) [36].

**Table 1:** Classification of comfort interventions by Comfort Theory

Categories	Definition	Examples
Technical	Interventions designed to maintain homeostasis and manage pain	Monitoring of vital signs, blood chemistries, administration of pain medications
Coaching	Interventions designed to relieve anxiety, provide reassurance and information, promote hope, listen and help plan realistically for recovery, integration or death in a culturally sensitive way	Emotional support, reassurance, education
Comfort food for the soul	Comfort measures that target transcendence, making patients feel strengthened in an intangible, personalised sort of way	Massage, guided imagery, music therapy

**Table 2:** Classification of NPIs by The Plateforme CEPS

Categories	Definition	Subcategories and examples
Psychological	That are potentially scalable include modified, evidence-based psychological treatments	Art Therapy: music therapy, dance therapy; health education programs; psychotherapies: cognitive behavioural therapy; mind–body programmes: yoga; animal-assisted therapies: zootherapies
Physical	Non-invasive and manualised program using passive or active mobilisation of the body	Exercise programs; horticultural therapies: garden therapies; physiotherapies: kinesitherapies; manual therapies: healing touch, massage, therapeutic touch; balneological programs
Nutritional	Related to improved infant, child and maternal health; stronger immune systems; safer pregnancy and childbirth; lower risk of non-communicable diseases; and longevity	Dietary supplementations; nutritional programs: diets
Digital	Evidence-based therapeutic interventions that are driven by high-quality software programs	M-health: eHealth devices, health apps; Healthcare videogames; virtual reality therapies;
Elemental	Non-invasive interventions using an elementary resource	Minerals; mycologicals: mushroom therapies; botanicals: herbal medicines; electromagneticals: hyperthermia therapies, light therapies; cosmeceuticals

According to Comfort Theory, effective comfort interventions can enhance individuals’ comfort and subsequently improve their HSBs, as well as the institutional integrity [4]. Some studies might evaluate participants’ comfort and/or related outcomes such as pain, anxiety and depression [28]. HSBs include three subcategories: internal behaviours, external behaviours and a peaceful death. The definition and examples of different types of outcome measures are presented in Table 3.

**Table 3:** The main outcome categories in the evidence map matrix

Outcomes categories	Definition	Examples
Comfort	The immediate experience of being strengthened through having the needs for relief, ease or transcendence met in four contexts: physical, psychospiritual, environmental and social contexts	Comfort level measured by any questionnaire or other approaches
Comfort-related variables	Close and relevant concepts to comfort experience	Pain, anxiety, depression, stress, fatigue
HSBs		
Internal behaviours	Occurring at the cellular or organ level, not visible from the outside, many indicators through lab work	Immune parameters, oxygen saturation, blood pressure, cardiac output
External behaviours	Observable behaviours	Ambulation, functional status, adherence to a medical regimen
A peaceful death	Dying and death with conflicts are resolved, symptoms are well managed, and acceptance by the patient and family members	A peaceful death, good death
Institutional integrity	The quality or state of health care organizations being complete, whole, sound, upright, professional and ethical providers of health care	Satisfaction, successful discharges, length of stay, financial stability of hospital

In the following, we will specify the criteria for determining the eligibility of references based on study design, interventions, population, settings and outcomes (PICOS framework).

### Dimensions

#### Types of study design

This EGM review will include impact evaluations and systematic reviews. Impact evaluations use one of the following designs: (1) randomised controlled trials (RCT), (2) quasi-experimental design, (3) before versus after studies and (4) observational description of services or practices. Systematic review (SR) refers to the review of primary studies adopting systematic approach, which includes (a) search of at least three databases, (b) screening with explicit inclusion criteria and (c) coding and reporting of all relevant findings [31], such as (1) systematic reviews, (2) scoping review and (3) integrative review. Both completed and ongoing studies (the study protocol) will be considered. This review will not include literature reviews (i.e. narrative reviews), qualitative studies, cohort studies and cross-sectional studies.

#### Types of population

The review will include adult participants who aged 18 and older, of any gender, race and ethnicity. To be inclusive, studies or reviews which state a focus on adults without providing the age of participants or that serve both adults and non-adults will be included. Participants groups may be but will not be limited to patients, their family members and HCPs.

#### Types of contexts

Participants can be from any geographic location and any settings as defined by the World Health Organization regions (WHO, African Region, Regions of the Americas, South-East Asian Region, European Region, Eastern Mediterranean Region, Western Pacific Region) [37]. The study context is not limited to any particular countries or health systems while it has to be in healthcare settings where all the intervention activities whose primary purpose is to promote, restore, or maintain health, such as hospital units, care facilities and home. Primary studies and systematic reviews that do not report the countries or settings will not be excluded.

#### Types of interventions

The review will include any interventions applying Kolcaba’s Comfort Theory to enhance comfort. The application can be that the Comfort Theory was used as the theoretical framework underpinned the intervention. Alternatively comfort questionnaires derived from the Comfort Theory and TS were used to measure participants’ comfort level. This review will only consider papers that clearly indicate that the Comfort Theory of Kolcaba or related comfort questionnaires were used, with cited references of which Dr Katherine Kolcaba was listed as the author or one of the authors.

As outlined in the conceptual framework above (Figs. 1 and 3), this EGM mapping review will develop the rows of the evidence map matrix with two classifications of interventions: (1) the typology of comfort measures based on Comfort Theory (Table 1) [4], and (2) the classification of PIs and NPIs by The Plateforme CEPS (Table 2) [36]. One included intervention could be categorised into both classifications. For example, hand massage will be coded as ‘comfort food for the soul’ in Comfort Theory typology whereas massage will also be categorised as an NPI.

#### Types of outcome measures

The columns of the evidence map matrix will be the main outcome categories that were developed by the authors based on the Comfort Theory, comprising (1) comfort, (2) comfort-related variables (added by authors), (3) HSBs and (4) institutional integrity. HSBs include three subcategories: internal behaviours, external behaviours and peaceful death. The main outcome categories and their definitions are presented in Table 3. Studies that did not measure comfort or comfort-related variables will be excluded as this is the focus of the review.

### Search methods and sources

A comprehensive search of eligible evidence will be conducted based on the framework of interventions and outcomes as outlined above, following the guidance by Campbell [30]. First, relevant studies will be identified through searches in electronic databases, including MEDLINE, CINAHL, PsycINFO, Embase, AMED, Web of Science, Scopus, CNKI (China National Knowledge Infrastructure), Wan Fang, Cochrane Library and JBI Library of Systematic Reviews. Second, grey literature will also be sought from Google Scholar, Baidu Scholar and The Comfort Line (the website disseminating Kolcaba’s Comfort theory). Thirdly, the reference lists of included papers will be chased and checked for additional sources. Lastly, key authors of primary studies or reviews will be contacted for information about unpublished work or on-going studies. The search process will be clearly documented for validation and transparency. The full search strategy for MEDLINE (EBSCO) is presented in Additional file 1 and which will be adapted to other databases or sources.

Papers written in English and Chinese will be considered for inclusion as the research team is proficient in the two languages. Most papers published in the widely used international databases are written in English so that the consideration of papers in English allows the most extent of coverage on papers met the inclusion criteria. Databases mainly covering publications in Chinese will be searched to scope evidence from the context of China. Papers published between 1991 and 2023 will be included as the first publication regarding Comfort Theory by Kolcaba was in 1991 [14, 27].

### Screening and study selection

Following the search, all identified papers will be imported into the software Endnote X9 (Clarivate Analytics, PA, USA). After removing duplicates, two reviewers will initially screen the title and abstract of each paper against the inclusion criteria on adult population, application of Comfort Theory, intervention types and outcome measures, language and publication year and exclude those that are considered completely irrelevant. Following the screening of title and abstract, full text of potentially relevant papers will be retrieved and reviewed in detail in qualitative data analysis software NVivo (QSR International, MA, USA) by two reviewers independently. Any disagreements that arise between the two reviewers at each stage of the study selection process will be solved through discussion with the third reviewer to achieve a final consensus.

The process and criteria of screening and selection will be piloted by the reviewers. Results of search and process of paper selection will be documented and presented in a PRISMA flow diagram [34].

### Data extraction and management

Each included study will be coded and extracted by two reviewers independently in NVivo software using the standardised coding form which has been piloted and refined (see Additional file 2). Discrepancies of data extraction will be discussed on regular meetings with the third reviewer to achieve consensus. The coding form was developed by the authors based on the framework of comfort interventions and outcomes, and which has been revised and refined after piloting on 20 relevant papers. In addition, details on characteristics of studies will be collected as filters for the evidence, such as population, country, settings and study design.

Once coding is completed, the univariate tabulations of all codes will be checked to identify uncoded data, out of range of codes and unexpected patterns in the data. All codes in a random sample of coded records will be systematically checked to find any code which seems to be used incorrectly.

### Assessing risk of bias and study quality

The methodological quality of systematic reviews will be appraised using AMSTAR-2 tool [38]. The tool JBI Critical Appraisal Checklist for Systematic Reviews and Research Syntheses will be used to assess the quality of scoping reviews and integrative reviews and other types of systematic reviews [39]. We will not assess the quality of included primary studies but study design of primary studies will be coded and included as a filter as per the guidance for evidence maps [31].

Quality appraisal of systematic reviews will be performed independently by two reviewers after which a consensus coding will be agreed upon. In case of disagreements that cannot be reconciled between the two reviewers, a third reviewer will make the final assessment.

### Analysis and presentation

If multiple reports exist for the same study, for example, both working papers and journal articles, the latest or most complete version will be used in the map. If different papers report different analyses, for example on different outcomes or for different subgroups, each paper will be included. In a publication with multiple studies, each eligible study will be shown in the map separately, meaning that a study with multiple interventions or outcomes will be shown multiple times on the map. Systematic reviews will be mapped based on question defined in the systematic review. Primary studies included will be mapped as well regardless of whether they are included in one or more systematic reviews.

The number of sources screened, assessed for eligibility, included and excluded will be presented in a PRISMA flow diagram. The EPPI-Mapper software, powered by EPPI-Reviewer [40], will be used to generate an online, interactive map of evidence about the international interventions applying Comfort Theory. The interventions and outcomes will make up the primary dimensions of the map. Each cell in the map will show studies and reviews which contain evidence on that combination of primary dimensions or absolute gaps where no evidence exists. The number of primary studies or included studies in a review will be shown by the size of the bubble on the map whereas the critical appraisal shown by colour of the bubble. In addition to interventions and outcomes, the following filters will be coded for primary studies and reviews where appropriate: types of theory application, study design (RCT, SR), population subgroups, WHO regions and countries, settings (intensive care unit (ICU), palliative care units, etc.), outcome measure tools or instruments related to Comfort Theory by Kolcaba (GCQ, etc.), publication information (type, year and language).

## Discussion

To the best of our knowledge, this EGM will be the first mapping review to identify the available evidence of the effects of interventions using Kolcaba’s Comfort Theory to promote adults’ comfort within an international scope. Findings from this EGM study can be used by researchers, policy-makers and practitioners from any disciplines and contexts aiming to enhance comfort. Further research will also be informed based on the findings of the EGM about the major gaps in the evidence base.

The important role of theory in developing complex interventions has been articulated by international guidelines [41, 42]. Comfort care falls into the group of complex interventions because comfort is difficult to measure, and consequently, it is difficult to develop effective comfort interventions. A theory clarifies how change is brought about, including the interplay of mechanisms and context. More informed use of theory can strengthen improvement programmes and facilitate the evaluation of their effectiveness [43]. Kolcaba’s Comfort Theory as the most widely used theory in relation to comfort should become one of the choices for developing and testing interventions and measurements in comfort care so that the effectiveness and quality of care can be ensured.

A large number of diverse papers is expected to be retrieved, coded and presented from this EGM which is reasonable for a middle-range theory existing for more than three decades. Potential challenges will be addressed by the three reviewers’ joint efforts within a sufficient long period of time, guided by a standardised systematic methodology by Campbell EGM [31].

## Supplementary Information

**Additional file 1.** Search strategy in MEDLINE (EBSCO).

**Additional file 2.** Coding form.

## Data Availability

Not applicable.

## Abbreviations

AMED: The Allied and Complementary Medicine Database
CINAHL: Cumulative Index to Nursing and Allied Health Literature
CNKI: China National Knowledge Infrastructure
EGM: Evidence and gap map
Embase: Excerpta Medica Database
EoL: End-of-life
GCQ: General Comfort Questionnaire
HCPs: Healthcare professionals
HSBs: Health-seeking behaviours
ICU: Intensive care unit
JBI: Joanna Briggs Institute
NPIs: Non-pharmacological interventions
PICOS: Population, Interventions, Comparison, Outcomes and Study Designs
PIs: Pharmacological interventions
PRISMA-ScR: Preferred Reporting Items for Systematic reviews and Meta-Analyses extension for Scoping Reviews
RCT: Randomised controlled trial
SR: Systematic review
The Plateforme CEPS: The Collaborative University Platform for Evaluating Health Prevention and Supportive Care Programs
TS: Taxonomic structure
WHO: World Health Organization
