# Supplementary material for: Interventions and practices using Comfort Theory of Kolcaba to promote adults’ comfort: an evidence and gap map protocol of international effectiveness studies
Source: Syst Rev. 2023 Mar 6;12:33. doi: 10.1186/s13643-023-02202-8 (PMC9987143; doi:10.1186/s13643-023-02202-8)
Supplement: Supplementary file 1 — Additional file 1. Search strategy in MEDLINE (EBSCO). [file 13643_2023_2202_MOESM1_ESM.pdf]

Search strategy in MEDLINE (EBSCO)

PICO	Search terms
P (Population)	Limiters Age: young adult:19-24 years, - aged,80 and over, aged: 65+years, adult: 19-44 years, middle aged:45-64years, all adult: 19+years
I (Interventions)	Comfort Theory, Theory of Comfort, Kolcaba, Kolcaba's theory, Kolcaba's theory of comfort, Kolcaba's Comfort Theory, Comfort Theory of Kolcaba, Holistic Comfort Theory, Theory of Holistic Comfort, Comfort care, Comfort intervention*, Comfort promotion*, Comfort enhancement*, Comfort practice*, Application* of Comfort Theory
C (Comparison)	
O (Outcomes)	Holistic comfort, Comfort assessment, Comfort measurement, Comfort evaluation, Comfort questionnaire, Comfort scale, Patient Comfort
Limiters	Date of Publication:1991- 2023<br>Narrow by Language: Chinese, English
