# Supplementary material for: Interventions and practices using Comfort Theory of Kolcaba to promote adults’ comfort: an evidence and gap map protocol of international effectiveness studies
Source: Syst Rev. 2023 Mar 6;12:33. doi: 10.1186/s13643-023-02202-8 (PMC9987143; doi:10.1186/s13643-023-02202-8)
Supplement: Supplementary file 2 — Additional file 2. Coding form. [file 13643_2023_2202_MOESM2_ESM.pdf]

Coding Form

**Population**	
**Types of group**	Patients with diseases, or conditions, family members, HCPs, health general people (students), other groups
**Age**	Young adults, middle-aged adults, old adults
**Gender**	Female, male, non-binary, not reported
**Region and country**	
**WHO region**	Asia, Europe, North America, South America, Oceania, Antarctica;
**Country**	Specify: USA, China, UK, Australia, etc.
**Settings**	Hospital units, home, community, long term care facility/ assisted living facility/nursing home, hospice, clinic, university, setting not reported
**Hospital units**	ICU, palliative care unit, emergency department etc.
**Theory application**	Theoretical framework for interventions, instruments assessing comfort outcome; using both theoretical framework and instruments, other types of application
**Study design**	RCT, quasi-experimental, service description/observational study, systematic review, scoping review, integrative review, other matching design
**Publication language**	English, Chinese
**Publication year**	1991-2000, 2001-2010, 2011-2023
**Interventions**	
**PIs**	Opioids, surgery, etc.
**NPIs**	Physical NPIs (art therapies, health education programs etc.), Nutritional NPIs, Digital NPIs (m-health etc.), Elemental NPIs (minerals, botanicals etc.)
**Comfort measures**	Technical comfort measures (monitoring of vital signs, blood chemistries, administration of pain medications), Coaching (emotional support, reassurance, education, listening), Comfort food for the soul (massage, guided imagery, music therapy)
**Outcomes**	
**Comfort**	comfort
**Comfort related variables**	pain, anxiety, depression, stress, fatigue
**Health seeking behaviors**	external (healing, immune function), internal (self-care, rehabilitation), a peaceful death
**Institutional integrity**	satisfaction, successful discharges, length of stay, costs
